# Instrument-supported gait analysis characterizes gait domain changes in patients with suspected normal pressure hydrocephalus

**DOI:** 10.1186/s42466-025-00394-z

**Published:** 2025-06-16

**Authors:** Carolin Semmler, Veronika Wunderle, Taylan D. Kuzu, Oezguer A. Onur, Christian Grefkes, Michael T. Barbe, Gereon R. Fink, Peter H. Weiss

**Affiliations:** 1 Faculty of Medicine, University of Cologne, Cologne, Germany; 2 Department of Neurology, University Hospital Cologne, Cologne, Germany; 3 Department of Neurology, University Hospital, Goethe University Frankfurt, Frankfurt (Main), Germany; 4 Institute of Neuroscience and Medicine (INM-3), Cognitive Neuroscience, Forschungszentrum Jülich, Jülich, Germany

**Keywords:** iNPH, Center of force, Center of pressure, Ground reaction force, Kinetics

## Abstract

**Background:**

Idiopathic Normal Pressure Hydrocephalus (iNPH) is a potentially reversible cause of cognitive impairment, urinary incontinence, and gait disturbances, which typically present with a characteristic slow, shuffling, and wide-based gait. Gait velocity, which is reduced relative to healthy controls, improves in iNPH patients following a spinal tap test. This study aimed at evaluating the criterion of a 20% gait velocity improvement in the 10 m walk test to identify responders and non-responders in a cohort of patients with probable iNPH receiving a spinal tap test as well as the added value of instrument-supported gait analysis.

**Methods:**

We assessed *pace*, *rhythm*, *variability*, *postural control*, and *force* in 59 patients with clinically suspected iNPH undergoing a spinal tap test, applying the 10 m walk test and an instrument-supported gait analysis. The change in gait velocity assessed in the 10 m walk test was used to differentiate patients with a positive response to the spinal tap (> 20% improvement, responders) from those with no relevant response (< 20% improvement, non-responders). Group differences were analyzed using chi-square tests, independent sample t-tests, Mann–Whitney-U tests and repeated measure ANOVAs.

**Results:**

Unlike non-responders (n = 39), responders (n = 20) showed significant changes in the gait domain *pace* in the 10 m walk test. Moreover, instrument-supported gait analyses revealed additional improvements in the gait domains *variability, rhythm, postural control* and *force* in responders only.

**Interpretation:**

This study confirmed the clinical utility of the 20% gait velocity improvement criterion for differentiating responders and non-responders in a cohort of patients with mostly probable iNPH, in whom clinical parameters alone were insufficient for classification. Notably, instrument-supported gait analysis validated this criterion by providing a more comprehensive characterization of gait disturbances compared to the 10 m walk test. However, further—especially longitudinal—studies are needed to reveal the full potential of the instrument-supported gait analysis in patients with (early/probable) iNPH.

**Supplementary Information:**

The online version contains supplementary material available at 10.1186/s42466-025-00394-z.

## Background

Idiopathic normal pressure hydrocephalus (iNPH) was first described in 1965 by Hakim and Adams [1]. Characteristic iNPH symptoms are subsumed as Hakim’s Triad, comprising gait disturbance, cognitive impairment, and urinary incontinence [2]. In most patients, the gait disturbance is the first symptom, initially perceived as an insecure gait or imbalance [3]. Subsequently, the gait worsens, gradually becoming slower and broad-based [4] with decreased step length [5] and outward-rotated feet6. These gait disturbances increase the risk for falls and thus reduce the mobility and quality of life of patients with iNPH [7–10].

CSF drainage via a ventriculoperitoneal shunt constitutes the standard iNPH treatment, often leading to long-term symptom improvement, particularly in gait [11]. In order to preoperatively predict the patients’ response to a ventriculoperitoneal shunt, but also to support the clinical diagnosis of an iNPH, a spinal tap test is performed [12]. While gait disturbances in patients with iNPH typically improve after 30–50 ml CSF drainage, it is still debated which gait parameters are best suited to quantify a patient’s spinal tap test response. The 10 m walk test, allows a time-effective recording of spatiotemporal parameters, e.g., the number of steps, turning steps, and walking time, also enabling to calculate walking speed [13]. After a spinal tap test, iNPH patients commonly demonstrate increased gait velocity [6, 14, 15] and a reduced number of steps and turning steps [9, 16]. However, the assessment of gait pattern changes by the 10 m walk test is limited to effects observable with the naked eye, neglecting undetected albeit informative alterations in not directly observable gait domains.

Lord and colleagues [17] characterized four gait domains: *pace*, *rhythm*, *variability,* and *postural control*. The gait changes of iNPH patients after spinal tap can be predominantly assigned to the gait domain *pace*. In particular, Stolze and colleagues [6] identified an improvement in gait velocity of at least 20% as the most sensitive parameter for spinal tap test responsiveness [6]. This finding was supported by further studies [18–20]. Furthermore, studies that analyzed additional spatiotemporal parameters in iNPH patients by instrument-supported gait analyses showed changes in the gait domains *variability* and *postural control* after a spinal tap [17], as iNPH patients reduced their stride time variability [21] and decreased their gait width [6, 15, 16]. Improvement in the gait domain *rhythm *[17] could be observed for gait cycle phases since iNPH patients’ showed a reduced double limb support phase [6, 15] and stance phase duration, as well as a respective increase in their swing phase duration after spinal tap. In contrast, gait parameters without significant responses to a spinal tap were foot rotation [6] and kinematic parameters, including the flexion and extension angles of knees and hips during walking [16] and foot-to-floor clearance [6, 16]. Moreover, changes in stride length [6, 14–16] and stride cadence [6, 14, 15] are controversially discussed in previous reports.

Therefore, we aimed to significantly advance the understanding of gait disturbances in iNPH by analyzing five different gait domains, including kinetics and balance parameters. This is the first study applying the criterion of 20% gait velocity improvement to extensively analyze the spinal tap test response of a large cohort of patients with probable iNPH before diagnosis and differentiating responders and non-responders by comprehensive gait and balance assessments, including kinetics.

## Methods

### Participants and study design

The patients with suspected iNPH included in this retrospective study were admitted to the Department of Neurology of the University Hospital Cologne for clinical evaluation, including an instrument-supported gait analysis. The study population consisted of 59 in-patients (39 male) with a mean age of 75.6 years (SD = 6.6, range: 60–91 years). The study was conducted under the Declaration of Helsinki and authorized by the local ethics committee (Cologne, study no.: 24–1065).

### Inclusion criteria

Patients were included in this study if they were aged between 60 and 95 years and suspected to suffer from iNPH based on clinical criteria, including gait disturbance, cognitive decline, urinary incontinence, and abnormalities in structural brain imaging consistent with iNPH [1]. Notably, these criteria are often less clear in clinical practice, especially in early iNPH stages.

A clinically observed gait disturbance was presented in all 59 patients (100%). In contrast, urinary incontinence was diagnosed in 41 patients (69.5%), and cognitive impairment was established in 40 patients (67.8%), based on DemTect scores [22]. 50 patients (84.7%) presented with ventricular enlargement in MRI or CT imaging [23]. In summary, 56 patients (94.9%) fulfilled the criteria for a probable iNPH based on iNPH guidelines (i.e., gait disorder plus urinary incontinence and/or cognitive impairment) [24].

Exclusion criteria were a history of strokes, brain tumors, neuropsychiatric disorders, and already existing ventriculoperitoneal shunts. All data used in this study were acquired during the routine clinical evaluation of the patients with suspected iNPH.

### Gait- and balance analysis

#### Equipment

Gait and balance assessments were performed using ground reaction force plates and pressure distribution platforms. A Leonardo Mechanograph® Gangway (henceforth called gangway; Novotec Medical GmbH, Pforzheim, Germany, 600 × 77 cm, 400 Hz) was used to measure center of force, while a Leonardo Mechanograph® Ground Reaction Force Plate (henceforth called force plate; Novotec Medical GmbH, Pforzheim, Germany, 66 × 66 cm, 800 Hz) assessed forces during standing and sitting balance tasks. For the latter, a modular bench (46 cm) was used. The data were analyzed with the Leonardo Mechanography® software (Version 4.4b06.29). Additionally, two zebris® FDM platforms (300 × 60.5 cm, 50 Hz) measured pressure distribution data (henceforth called pressure walkway; zebris Medical Systems, Tübingen, Germany). These data were analyzed with the zebris FDM software (Version 1.18.48).

#### Procedure

##### Clinical 10 m walk test

Patients performed the 10 m walk test in their preferred gait velocity, involving a 180° turn [16], while the number of steps, the time required for walking 20 m (= 2 × 10 m) and the number of turning steps were recorded. From these, gait velocity and cadence (i.e., stride frequency) were calculated.

##### Balance and walk tests on the force and pressure plates

For the standing balance assessment, patients stood on the force plate with feet aligned, arms relaxed at their sides and eyes closed for 10 s, assessing the sway area and force variability.

In the sitting balance test, patients sat on the bench attached to the force plate with feet hip-width apart and hands resting on their thighs for 10 s with eyes closed. The sway area of the trunk and force variability were measured.

Next, the patients performed walk tests on the gangway (4 × 6 m = 24 m total) and pressure walkway (6 × 3 m = 18 m total) at their preferred gait velocity. The following gait parameters were calculated and assigned to the respective gait domain (adapted from Lord and colleagues [17]):

(i) *Pace*: gait velocity, number of steps per meter, step length
(ii) *Rhythm*: ratio of stance, swing, and double limb support phases about the gait cycle
(iii) *Variability*: intra-individual step length variability
(iv) *Postural control*: gait width, path length per distance, and foot rotation
(v) *Force*: total maximum force and resulting power, both for the whole walk across the platforms and separately for the heel-strike and toe-off phase, height difference of the body’s center of gravity

All patients were measured before and after the spinal tap, wearing their own shoes to avoid altered gait patterns due to discomfort. Patients with severe gait disturbance were accompanied to prevent falling.

### Statistical analysis

All parameters were normalized to the patient’s height and weight. Per patient and time point (i.e., before and after spinal tap), an average of 45 steps (SD: 16.8) on the gangway and 33 steps (SD: 6.7) on the pressure walkway were analyzed. For statistical analysis, all parameters of a given patient were averaged for each instruments across all walks per time point (i.e., before and after spinal tap).

To compare parameters before and after the spinal tap, paired samples t-tests and Wilcoxon signed-rank tests were used.

Responders were classified based on an improvement of at least 20% in their gait velocity after the spinal tap measured in the 10 m walk test. Differences between responders and non-responders were analyzed using chi-square tests, Mann–Whitney-U-tests, and repeated measures ANOVAs with the within-subject factor time (i.e., before and after spinal tap) and the between-subject factor group (i.e., responders vs. non-responders). Results were corrected for multiple comparison using the Benjamini–Hochberg method [25]. A potential association between the time interval between the spinal tap and the subsequent gait assessment and the patient’s response to the spinal tap was examined using Spearman correlation. Additionally, a potential difference in this time interval between responders and non-responders was analyzed using a Mann–Whitney-U test. A significance level of α = 0.05 was set for all statistical analyses. For easier readability of the figures, change scores were inverted for parameters, in which a reduction indicates relative improvement compared to baseline performance (e.g., number of steps per meter), denoted as improvement scores.

## Results

### Group allocation of patients

Twenty patients improved their gait velocity by at least 20% in the 10 m walk test and were classified as responders, while 39 patients did not fulfill this criterion and were classified as non-responders. Demographic details are presented in Table 1. There were no significant group differences regarding age, body weight, body height, or sex distribution (all *p* > 0.290). The duration between spinal tap and the subsequent gait assessment (median: 1 day, IQR: 1–7 days) did not differ between groups (*p* = 0.185) nor was it significantly associated with patient’s response to the spinal tap across groups (*p* = 0.792). Regarding clinical symptoms, no significant association was found between group allocation (i.e., responders and non-responders) and probable iNPH criteria according to guidelines (χ^2^ = 1.01, df = 1, *p* = 0.315). Thus, the proportion of patients fulfilling the criteria for probable iNPH was not significantly different in the groups of responders (n = 19/20) and non-responders (n = 37/39). The proportion of patients with ventricular enlargement in clinical MRI or CT imaging did not differ between the two groups (responders: 19/20 non-responders: 31/39; χ^2^ = 1.06, df = 1, *p* = 0.303). Similarly, no significant association was found between the group allocation and the presence of the clinical symptoms contributing to the iNPH criteria, i.e., urinary incontinence (responders: 16/20, non-responders: 25/39; χ^2^ = 2.07, df = 1, *p* = 0.150) and cognitive impairment (responders: 13/20, non-responders: 27/39; χ^2^ = 0.00, df = 1, *p* = 0.953). Finally, there was no significant difference in the Fazekas scores between responding and non-responding patients for whom MR imaging was available (responders (n = 14): Md (IQR) = 2 (2.5–1.5); non-responders (n = 19): Md (IQR) = 1.5 (2.5–0.5); U = 101.5, Z = − 1.162, *p* = 0.245).

**Table 1:** Demographic information of patients with suspected iNPH who responded (responders) or did not respond (non-responders) to a spinal tap

		Responders n = 20	Non-responders n = 39	*p*-value
Age (years)	M (SD)	75.0 (7.4)	76.0 (6.2)	0.485
Sex	male/female	14/6	25/14	0.651
Body weight (kg)	M (SD)	81.9 (15.4)	79.7 (15.7)	0.968
Body height (cm)	M (SD)	169.2 (8.4)	170.9 (9.1)	0.290

### Performance in the 10 m walk test

**Fig. 1:**
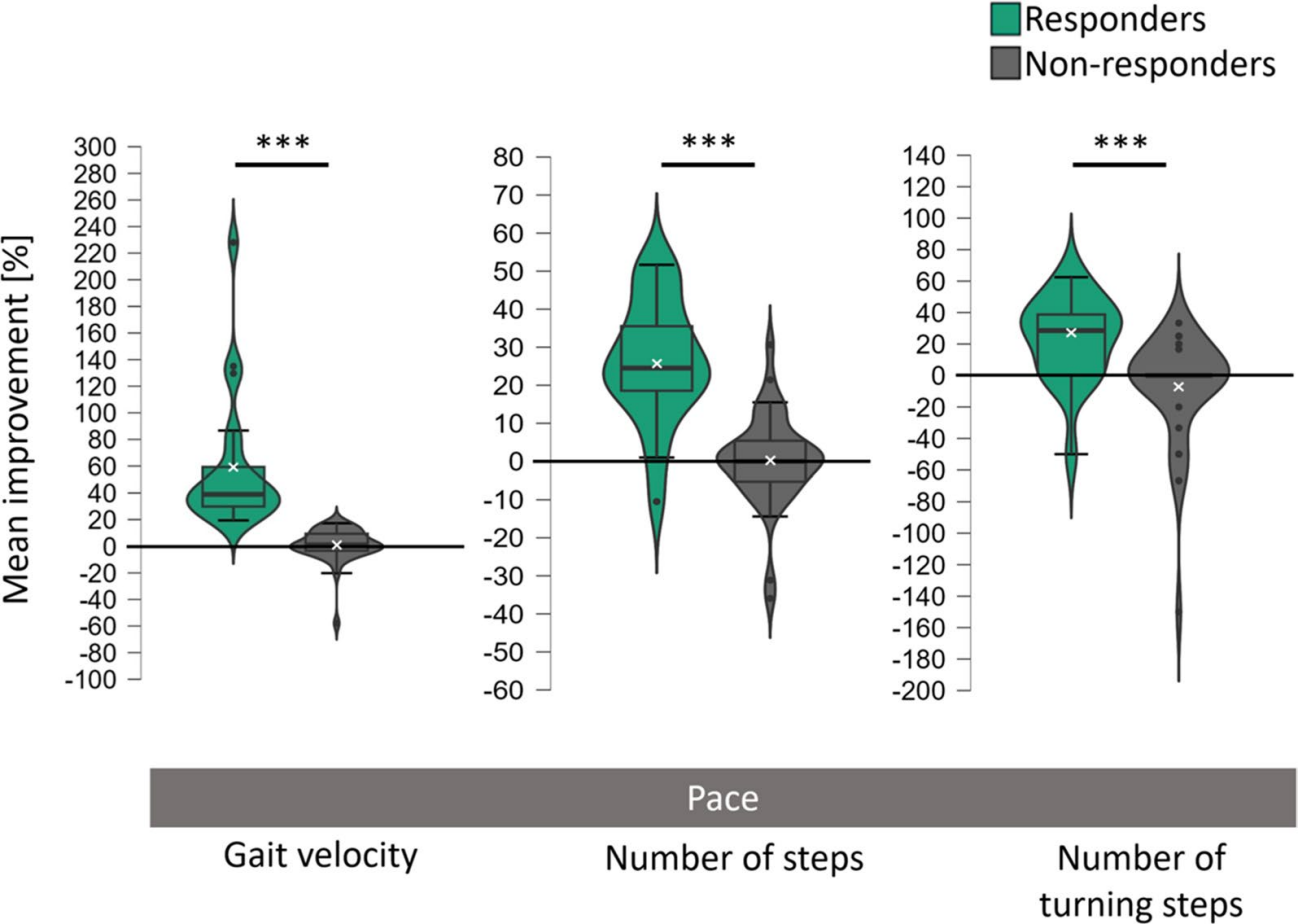
Mean improvement scores of responders and non-responders in the 10 m walk test. Improvement scores (in percent) were calculated relative to baseline values and were inverted for parameters, in which a reduction indicates relative improvement compared to baseline performance (e.g., number of steps). The grey bar indicates the assessed gait domain, here: pace = gait domain of pace. The white ‘x’ marks the respective groups’ mean scores. All p-values are FDR-corrected. *** = *p* < 0.001

Results of within and between group comparisons are depicted in Fig. 1 and Table 2. Results of the main effects of group and time of the repeated measures ANOVA are reported in Table S1 of the supplementary material.

**Table 2:**
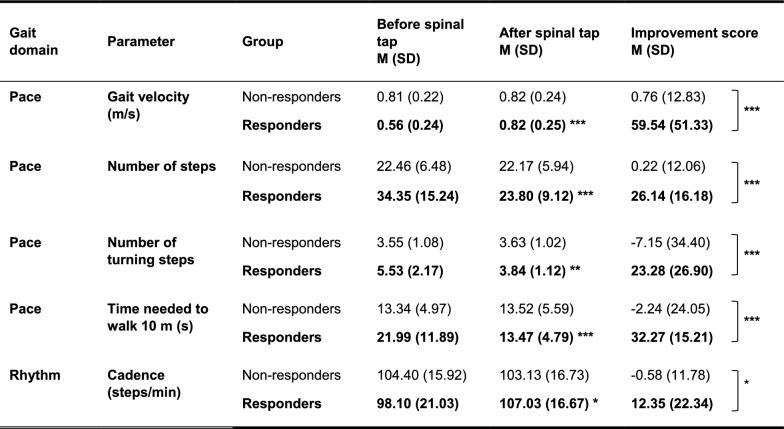
Mean scores of responders and non-responders in the 10 m walk test

Non-responders n = 39, Responders n = 20. Improvement scores are calculated relative to baseline values and were inverted for parameters where a reduction indicates relative improvement compared to baseline performance. Gait domains/ parameters in which the responders showed a significant improvement after spinal tap are marked in bold. All p-values are FDR-corrected. * = *p* < 0.05, ** = *p* < 0.01, *** = *p* < 0.001

Responders significantly improved in the gait domain *pace*, showing an increased gait velocity (+ 59.5%, p_FDR_ < 0.001), reduced walking time (− 32.3%, p_FDR_ < 0.001), fewer steps (− 26.1%, p_FDR_ < 0.001) and turning steps (− 23.3%, p_FDR_ = 0.002). Non-responders showed no significant changes in these parameters (all p_FDR_ > 0.353). Consequently, significant interaction effects between the factors time and group were identified for the gait velocity (*F*(1, 57) = 76.00, p_FDR_ < 0.001, partial η^2^ = 0.57), number of steps (F(1, 57) = 41.74, p_FDR_ < 0.001, partial η^2^ = 0.42), walking time (F(1, 57) = 32.81, p_FDR_ < 0.001, partial η^2^ = 0.37), the number of turning steps F(1, 55) = 26.14, p_FDR_ < 0.001, partial η^2^ = 0.32), and the stride cadence (*F*(1, 57) = 5.53, p_FDR_ < 0.05, partial η^2^ = 0.09).

### Instrument-supported gait and balance analyses

#### Balance test performances

No significant changes were observed in the balance tests for either group. Moreover, the parameters of the balance tests showed no significant interaction effect between the factors group and time (all p_FDR_ > 0.081).

#### Walk tests on force and pressure distribution walkways

##### Gait domain changes within groups after the spinal tap

**Fig. 2:**
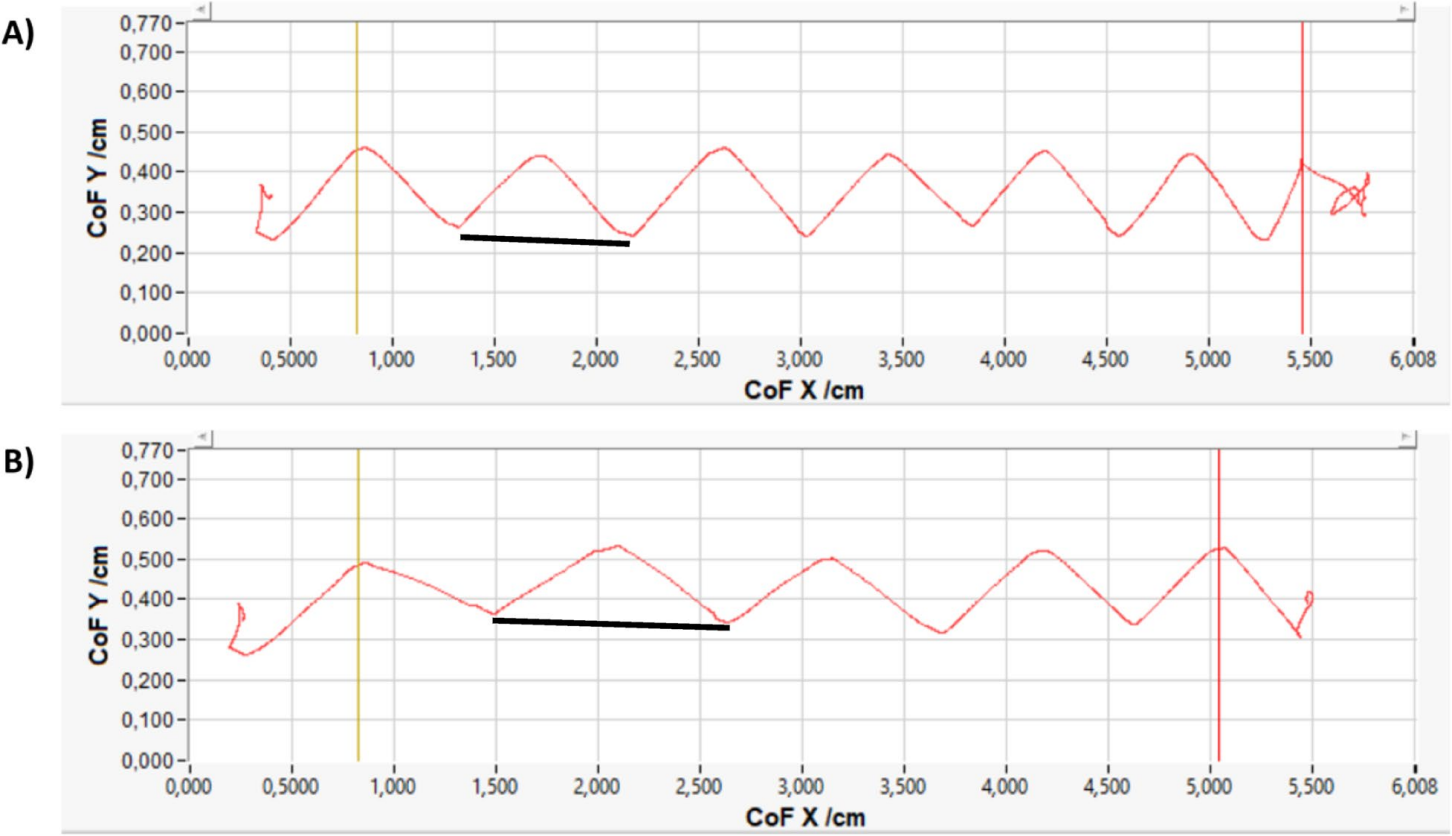
Path of a responders’ center of force depicted over time on the gangway. **A** Before spinal tap test. **B** After spinal tap test. The path of a responder’s center of force over time is shown before (**A**) and after (**B**) receiving a spinal tap test. The y-axis represents the width of the gangway, whereas the x-axis represents the length of the gangway. The red line illustrates the path of the patient’s center of force moving on the gangway over time. The zigzag pyramidal shapes of the path show the respective steps with the left and right leg and how the body’s center of force is moving along with the steps as the bodyweight shifts onto the respective leg. As one of the most prominent changes, the black horizontal line below one of the red pyramidal shapes marks the step length of the responder, which has significantly increased from before (**A**) to after spinal tap (**B**). Also, as seen in the total number of pyramidal shapes before and after the spinal tap, the number of steps has significantly decreased. The yellow vertical line marks the beginning of the analysis, hereby excluding the first step of the participant. In contrast, the red vertical line marks the end of the analysis, hereby excluding the last step of the participant

Consistent with results from the 10 m walk test, responders significantly improved in the gait domain *pace*, showing increased gait velocity (+ 29.9%, p_FDR_ < 0.001), fewer steps per meter (-28.6%, p_FDR_ < 0.001) and increased step length (+ 46.4%, p_FDR_ < 0.001) after the spinal tap (see Table 3). In Fig. 2, the exemplary paths of a responder’s center of force on the gangway before (see Fig. 2A) and after the spinal tap (see Fig. 2B) are depicted. Non-responders showed no significant changes in *pace* parameters (all p_FDR_ > 0.936).

**Table 3:**
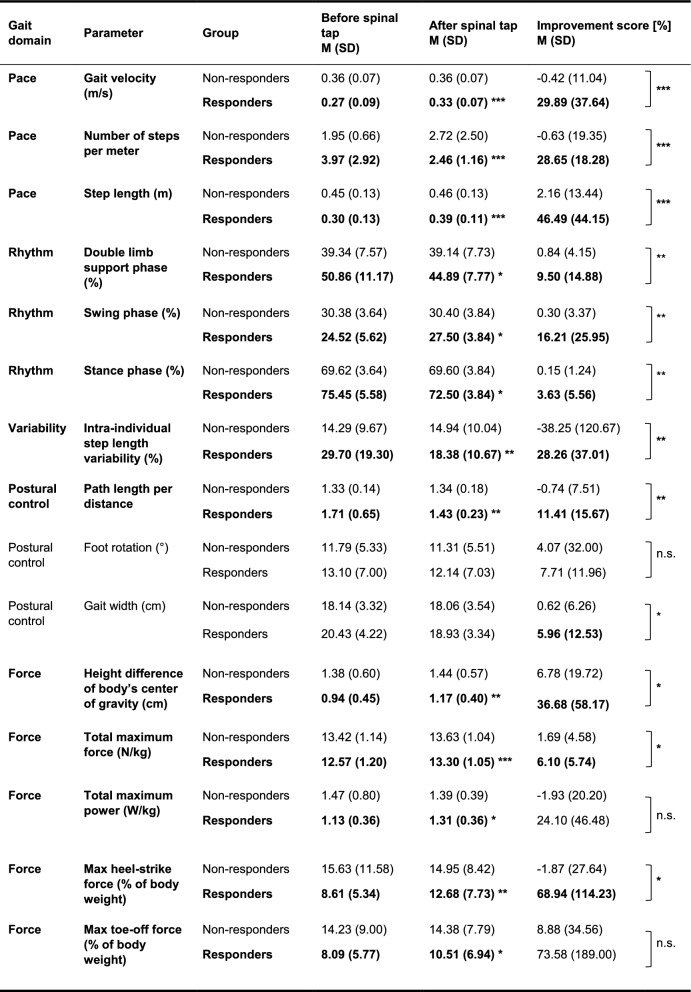
Changes in gait parameters of responders and non-responders assessed on force and pressure distribution walkways

Non-responders N = 39, Responders N = 20. Improvement scores are calculated relative to baseline values and were inverted for parameters where a reduction indicates relative improvement compared to baseline performance. Gait domains/ parameters in which the responders showed a significant improvement after spinal tab are marked in bold

All *p*-values are FDR-corrected. * = *p* < 0.05, ** = *p* < 0.01, *** = *p* < 0.001

In the gait domain *rhythm*, significant changes were observed in the gait cycle phases (i.e., double limb support phase, swing phase, and stance phase) for responders (− 9.5%, − 16.2% and + 3.63%, respectively; all p_FDR_ < 0.046), whereas non-responders showed no significant change in *rhythm* parameters (all p_FDR_ > 0.693). Moreover, responders significantly improved in the gait domain *variability* by significantly reducing their intra-individual step length variability (− 28.3%; p_FDR_ < 0.01), which was not the case in non-responders (p_FDR_ = 0.834). Responders also improved in the gait domain *postural control*, reducing their path length per distance (− 11.4%, p_FDR_ < 0.05), but did not significantly change their foot rotation or gait width (both p_FDR_ > 0.067). Parameters of the gait domain *postural control* did not significantly change in non-responders (all p_FDR_ > 0.900; Table 3).

Finally, responders improved their gait regarding the *force* domain by significantly increasing their total maximum force and resulting power, both for the whole walk across the platforms (+ 6.1% and + 24.1%, respectively, both p_FDR_ < 0.05) and separately for the heel-strike (+ 68.9%, *p* = 0.003) and toe-off moment (+ 73.6%, p_FDR_ = 0.041), leading to an increased height difference of their body’s center of gravity during walking (+ 36.7%, p_FDR_ = 0.002). Non-responders showed no significant changes in the gait domain *force* (all p_FDR_ > 0.085; Table 3).

##### Between-group comparison of gait domain changes after the spinal tap

**Fig. 3:**
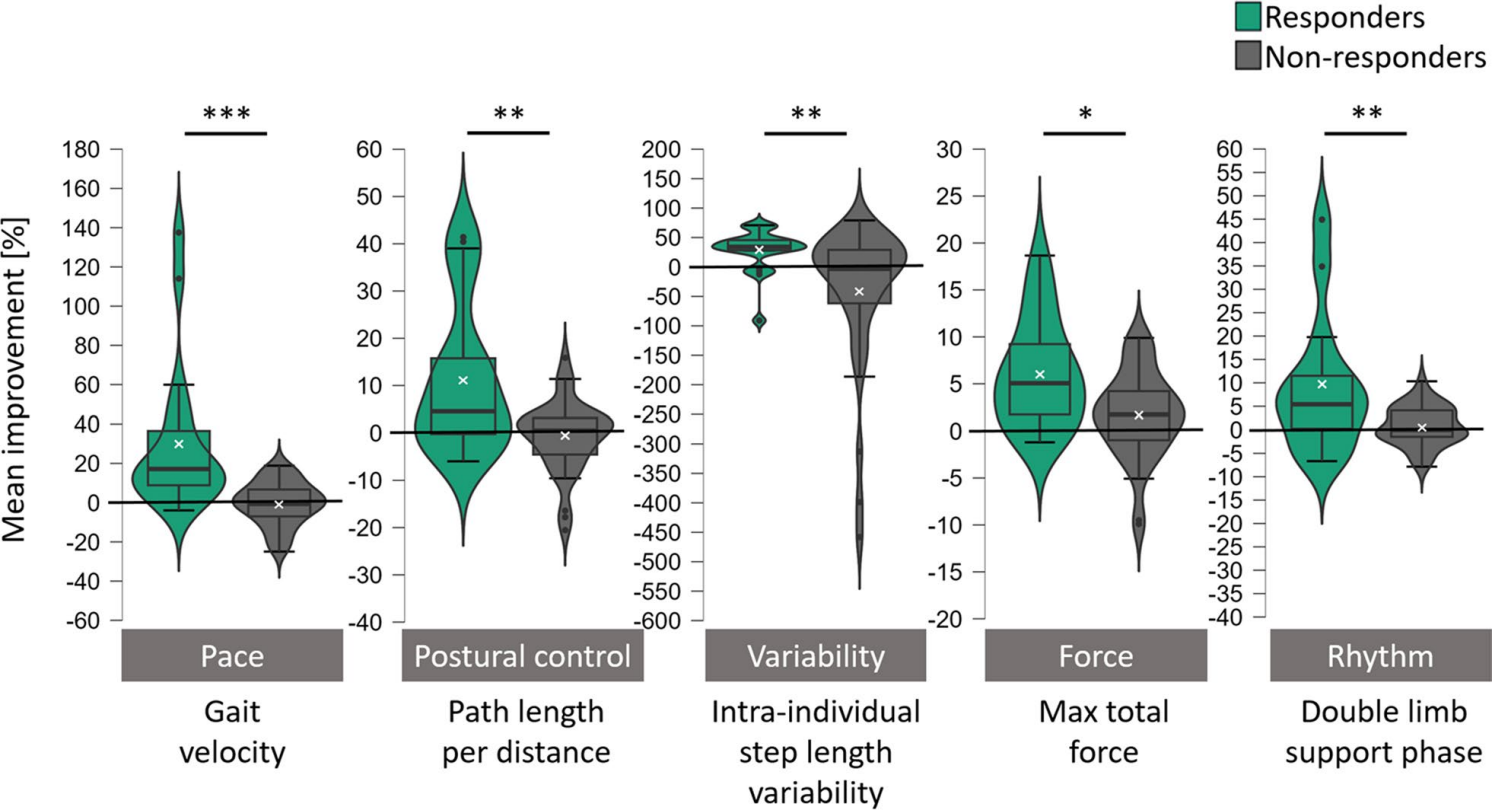
Mean improvement scores of responders and non-responders in the walk tests performed on force and pressure distribution walkways given in percent. Improvement scores were calculated relative to baseline values and were inverted for parameters where a reduction indicates relative improvement compared to baseline performance (e.g., path length per distance). The grey bars below each plot indicate the respective gait domain, e.g., Postural control = gait domain of *postural control*. The white ‘x’ marks the respective groups’ mean scores. All p-values are FDR-corrected. * =*p* < 0.05, ** = *p* < 0.01, *** = *p* < 0.001

In the *pace* domain, significant interaction effects between the factors time and group were identified for all parameters, including the gait velocity (*F*(1, 56) = 22.49, p_FDR_ < 0.001, partial η^2^ = 0.29), number of steps per meter (*F*(1, 56) = 12.73, p_FDR_ < 0.001, partial η^2^ = 0.19), and step length (F(1, 56) = 31.92, p_FDR_ < *0.001,* partial η^2^ = 0.36; see Fig. 3 and Table 3). Besides, for the parameters of the rhythm domain, significant interaction effects between the factors time and group were identified for the double limb support phase (*F*(1, 46) = 11.76, p_FDR_ < 0.005, partial η^2^ = 0.20), the swing phase (*F*(1, 46) = 12.51, p_FDR_ < 0.005, partial η^2^ = 0.21), and the stance phase (*F*(1, 46) = 12.54, p_FDR_ < 0.005, partial η^2^ = 0.21). In the *variability* domain, significant interaction effects between the factors time and group were identified for the intra-individual step length variability (*F*(1, 56) = 10.46, p_FDR_ < 0.005, partial η^2^ = 0.16). Furthermore, in the *postural control* domain, significant interaction effects between the factors time and group were identified for the path length per distance (*F*(1, 56) = 14.16, p_FDR_ < 0.01, partial η^2^ = 0.20) and the gait width (*F*(1, 46) = 6.09, p_FDR_ < 0.05, partial η^2^ = 0.12), but not for the foot rotation (*F*(1, 46) = 1.52, p_FDR_ = 0.223, partial η^2^ = 0.03; see Fig. 3, Table 3). Lastly, for most parameters in the *force* domain, significant interaction effects between the factors time and group were identified, e.g., for the height difference of the body’s center of gravity (*F*(1, 56) = 6.02, p_FDR_ < 0.05, partial η^2^ = 0.10), total maximum force (*F*(1, 56) = 8.31, p_FDR_ < 0.05, partial η^2^ = 0.13), and heel-strike force (*F*(1, 56) = 8.26, p_FDR_ < 0.05, partial η^2^ = 0.13). For the maximum toe-off force and maximum total power, no significant interaction effects between time and group were found (*F*(1, 56) = 3.87, p_FDR_ = 0.068, partial η^2^ = 0.07 and *F*(1, 56) = 2.43, p_FDR_ = 0.124, partial η^2^ = 0.04, respectively), likely due to the high variability in responders.

## Discussion

This study aimed at evaluating the criterion of a 20% gait velocity improvement in the 10 m walk test to identify responders and non-responders in a cohort of patients with probable iNPH receiving a spinal tap test as well as the added value of instrument-supported gait analysis. The 20% gait velocity improvement criterion [6] effectively differentiated responders from non-responders after a spinal tap. Importantly, instrument-supported gait analyses disclosed additional changes in multiple gait domains, further characterizing the iNPH gait profile and its response to a spinal tap.

All 59 patients were evaluated for suspected iNPH-related gait disturbances. Although 94.9% of patients met the criteria for probable iNPH according to Relkin and colleagues[26], the objective cut-off of at least 20% gait velocity improvement after the spinal tap test [6] effectively classified patients as responders (n = 20, 33.9%) and non-responders (n = 39, 66.1%) using the 10 m walk test. Importantly, this approach showed that the 20% criterion could identify responders even in a clinical sample with mostly probable iNPH patients, who could not be categorized as responders or non-responders using the clinical parameters that were available before the spinal tap, i.e., cognitive impairment, urinary incontinence, and imaging findings.

The current findings align with previous literature [19–21], demonstrating that gait velocity and step length improve after a spinal tap in responders. Importantly, instrument-supported gait analysis confirmed that these improvements are accompanied by a reduced step length variability, reflecting a more regular, secure, and dynamic gait in responders.

Furthermore, improvements in heel-strike and toe-off force and in the vertical displacement of the body’s center of gravity support the hypothesis that spinal tap-induced gait changes are not limited to temporal parameters but also extend to kinetics. The heel-strike, a relatively passive movement that depends on the height of the body’s center of gravity [27], and the toe-off force, key to powerful and dynamic gait [27], are crucial for adequate step length and, consequently, a higher gait velocity, demonstrating the synergistic interplay of spatiotemporal parameters and kinetics.

The observed improvements in the gait domain *postural control*, specifically the reduction in sway of the body’s center of gravity, reflect a more efficient walking pattern (Fig. 2). However, the lack of relevant changes in the foot (outward) rotation and gait width are consistent with the previous literature [4, 15, 16], suggesting that these iNPH characteristic parameters may be less sensitive to short-term CSF-related improvements.

The absence of effects on static balance supports the notion that CSF drainage primarily influences dynamic rather than static steady-state balance and postural control, reinforcing that balance is task-dependent rather than a “general ability” [28]. While previous studies have documented increased sway in iNPH patients compared to healthy individuals [29], research on balance improvements following a spinal tap remains scarce, highlighting the need for further investigations. Similarly, additional research is needed to better understand changes in the gait domain *rhythm*. The improvements in the double limb support, swing, and stance phases are consistent with previous studies reporting gait cycle changes after CSF drainage [6, 15]. However, although responders also showed improvements in cadence, the only parameter from the *rhythm* domain assessed in the 10 m walk test, the current literature remains inconclusive regarding its responsiveness to CSF drainage [6, 30, 31].

This study is the first that applied the criterion of 20% gait velocity improvement to differentiate responders and non-responders in a cohort of patients suffering from clinically observable gait disturbances. Previous studies have examined gait velocity improvements after a spinal tap in patients with suspected iNPH. However, none has explicitly used the 20% threshold as a criterion to categorize responders and non-responders in a cohort defined by clinical criteria (possible or probable iNPH). Thus, our study is the first to show that the 20% criterion is also applicable to differentiate responders and non-responders in a clinical sample in which 94.9% of the patients fulfilled the criteria for a probable iNPH based on iNPH guidelines and in which clinical symptoms were not suitable to differentiate the patient groups before the spinal tap. This highlights the need for objective gait assessment methods beyond clinical assessments.

Applying the 20% gait velocity improvement in the 10 m walk test consequently disclosed further changes in the domain *pace*, which, however, are not independent of the 20% gait velocity criterion used for patient response categorization in the current study. Instrument-supported gait analysis provided validation to further assess whether this criterion also reflects changes in other gait domains, revealing alterations in *rhythm*, *variability*, *postural control*, and *force*. Notably, the 10 m walk test showed limited insight into the gait domain *rhythm* by assessing the parameter cadence, showing a less pronounced yet significant change after the spinal tap in responders. In contrast, the instrument-supported gait analysis confirmed significant effects in the gait domain *rhythm*, as swing phase, stance phase, and double limb support phase all exhibited significant interaction effects, indicating a differential improvement in responders compared to non-responders as defined by the 20% criterion of the 10 m walk test. Even though most non-responders (94.9%) fulfilled the clinical criteria for a probable iNPH [24], gait patterns in non-responders did not show relevant changes after the spinal tap, suggesting that these patients potentially suffered from other etiologies causing gait disturbances. This indicates that the instrument-supported gait analysis provided valuable information to confirm—for the first time—the clinical utility of the 20% gait velocity improvement in the 10 m walk test to differentiate patients within a clinically defined patient sample, in which most patients fulfilled the criteria for probable iNPH.

A meta-analysis by Passaretti and colleagues [18] highlighted the need for better characterization of response parameters after the spinal tap in suspected iNPH patients. The instrument-supported gait analysis offers significant advantages over the 10 m walk test by providing a multidimensional assessment of gait comprising multiple gait parameters and domains. While the 10 m walk test primarily measures the gait domain *pace*, the instrument-supported gait analysis captures changes across multiple gait domains, including *pace*, *rhythm*, *variability*, *postural control*, and *force*, revealing their synergistic interplay. This data allows for a more detailed and systematic understanding of how gait patterns change in probable iNPH patients responding to a spinal tap. Notably, non-responders showed no relevant improvements in any parameter the instrument-supported gait analysis measured. Thus, instrument-supported gait analysis provides a more comprehensive characterization of gait changes and potentially identifies subtle improvements that are most likely not detectable in a standard assessment with the 10 m walk test. Moreover, this more nuanced characterization of iNPH gait profiles could be particularly relevant for longitudinal observations of gait changes, including those occurring after shunt surgery, thereby contributing to a more comprehensive understanding of disease progression and treatment response. Furthermore, the detailed and precise assessment by instrument-supported gait analysis may prove valuable for early iNPH detection. As a first symptom, patients often report reduced security or balance when walking [3]. These subtle gait disturbances are usually not observable in the clinical examination and are hard to objectify by the 10 m walk test. Instrument-supported gait analysis may be more sensitive in detecting these subtle gait disturbances by assessing multiple parameters of different gait domains. However, further—mainly longitudinal—studies are needed to reveal the full potential of the instrument-supported gait analysis in patients with (early/probable) iNPH.

Some limitations should be acknowledged. First, the lack of data on outcomes after potential future ventriculoperitoneal shunts or continuous external lumbar drainage leaves diagnostic uncertainty regarding iNPH. Consequently, classifying patients with suspected iNPH into responders and non-responders by relevant (i.e., > 20%) changes in gait velocity after spinal tap remains somewhat arbitrary, although widely accepted [6]. Next, all patients performed the walk tests at a self-selected speed. Schniepp and colleagues [32] investigated the effect of spinal taps on the gait of iNPH patients for different walking speeds. They concluded that walking at the individual maximum speed should be preferably assessed since high-velocity gait is less affected by motivational factors compared to gait at a self-selected speed. Therefore, the results of the current study should be complemented by future investigations assessing gait in patients with suspected iNPH who walk at their maximum speed. Furthermore, due to the study’s retrospective nature, patients walked in their own shoes. Although this impacts standardization, this approach ensured that patients walked as naturally and comfortably as possible, increasing the ecological validity of the current findings.

## Conclusions

To conclude, this study confirmed the clinical utility of the 20% gait velocity improvement criterion for differentiating responders and non-responders in a cohort of patients with mostly probable iNPH, in whom clinical parameters alone were insufficient for classification. Notably, instrument-supported gait analysis validated this criterion by providing a more comprehensive characterization of gait disturbances compared to the 10 m walk test, showing changes in all gait domains in responders and highlighting their synergistic interplay. Further—mainly longitudinal—studies are warranted to reveal the full potential of an instrument-supported gait analysis in patients with (early/probable) iNPH.

## Supplementary Information

Additional file 1.

## Data Availability

The datasets used and/or analysed during the current study are available from the corresponding author on reasonable request.

## Abbreviations

CSF: Cerebrospinal fluid
iNPH: Idiopathic normal pressure hydrocephalus
