# Supplementary material for: Instrument-supported gait analysis characterizes gait domain changes in patients with suspected normal pressure hydrocephalus
Source: Neurol Res Pract. 2025 Jun 16;7(1):41. doi: 10.1186/s42466-025-00394-z (PMC12168308; doi:10.1186/s42466-025-00394-z)
Supplement: Supplementary file 1 — Additional file 1. [file 42466_2025_394_MOESM1_ESM.docx]

Supplementary material for the manuscript “Instrument-supported gait analysis characterizes gait domain

changes in patients with suspected normal pressure hydrocephalus” by Semmler & Wunderle et al.

Table S1. Results of the repeated measures ANOVA for the main effects of the between-subject factor GROUP and the within-subject factor TIME.

| Test / Domain | Parameter | Group effect | Time effect |
| --- | --- | --- | --- |
| **Balance** **standing** | Standing ellipse | F(1, 54) = 1.87, p_FDR_ = .177, partial η² = .03 | F(1, 54) = 0.02 , p_FDR_ = .890, partial η² = .00 |
|  | Standing force SD | F(1, 54) = 3.38, p_FDR_ = .143, partial η² = .06 | F(1, 54) = 4.85, p_FDR_ = .064, partial η² = .08 |
| **Balance sitting** | Sitting ellipse | F(1, 53) = 0.02, p_FDR_ = .883, partial η² = .00 | F(1, 53) = 0.49, p_FDR_ = .978, partial η² = .01 |
|  | Sitting force SD | **F(1, 53) = 7.59, p_FDR_ = .016, partial η² = .13** | F(1, 53) = 0.13, p_FDR_ = .725, partial η² = .00 |
| **Pace**  (10-meter walk test) | Gait velocity | F(1, 57) = 3.89, p_FDR_ = .067, partial η² = .06 | **F(1, 57) = 80.62, p_FDR_ = .000, partial η² = .59** |
|  | Number of steps | **F(1, 57) = 6.54, p_FDR_ = .012, partial η² = .01** | **F(1, 57) = 40.99, p_FDR_ = .000, partial η² = .43** |
|  | Turning steps | **F(1, 57) = 11.22, p_FDR_ = .007, partial η² = .17** | **F(1, 57) = 21.66, p_FDR_ = .000, partial η² = .28** |
|  | Time needed to walk 10m | **F(1, 57) = 4.17, p_FDR_ = .024, partial η² = .05** | **F(1, 57) = 32.15, p_FDR_ = .000, partial η² = .37** |
| **Rhythm**  (10-meter walk test) | Cadence | F(1, 57) = 0.00, p_FDR_ = .778, partial η² = .00 | F(1, 57) = 2.29, p_FDR_ = .083, partial η² = .04 |
| **Pace**  (instrument-supported gait analysis) | Gait velocity | **F(1, 56) = 10.33, p_FDR_ = .003, partial η² = .16** | **F(1, 56) = 17.74, p_FDR_ = .000, partial η² = .24** |
|  | Number of steps per meter | F(1, 56) = 3.90, p_FDR_ = .053, partial η² = .07 | F(1, 56) = 1.34, p_FDR_ = .251, partial η² = .02 |
|  | Step length | **F(1, 56) = 10.63, p_FDR_ = .006, partial η² = .16** | **F(1, 56) = 44.17, p_FDR_ = .000, partial η² = .44** |
| **Rhythm**  (instrument-supported gait analysis) | Double limb support phase | **F(1, 46) = 11.76, p_FDR_ = .002, partial η² = .20** | **F(1, 46) = 14.90, p_FDR_ = .001, partial η² = .25** |
|  | Swing phase | **F(1, 46) = 12.43, p_FDR_ = .010, partial η² = .21** | **F(1, 46) = 14.39, p_FDR_ = .000, partial η² = .24** |
|  | Stance phase | **F(1, 46) = 12.35, p_FDR_ = .003, partial η² = .21** | **F(1, 46) = 14.45, p_FDR_ = .001, partial η² = .24** |
| **Variability**  (instrument-supported gait analysis) | Intra-individual step length variability | **F(1, 56) = 11.31, p_FDR_ = .001, partial η² = .17** | **F(1, 56) = 8.31, p_FDR_ = .006, partial η² = .13** |
| **Postural control** (instrument-supported gait analysis) | Path length per distance | **F(1, 56) = 9.51, p_FDR_ = .010, partial η² = .15** | **F(1, 56) = 12.35, p_FDR_ = .003, partial η² = .18** |
|  | Foot rotation | F(1, 46) = 0.40, p_FDR_ = .531, partial η² = .01 | **F(1, 46) = 5.18, p_FDR_ = .028, partial η² = .10** |
|  | Gait width | F(1, 46) = 2.04, p_FDR_ = .241, partial η² = .04 | **F(1, 46) = 8.35, p_FDR_ = .009, partial η² = .15** |
| **Force**  (instrument-supported gait analysis) | Height difference | F(1, 56) = 5.77, p_FDR_ = .098, partial η² = .09 | **F(1, 56) = 17.65, p_FDR_ = .000, partial η² = .24** |
|  | Max force | F(1, 56) = 4.01, p_FDR_ = .084, partial η² = .07 | **F(1, 56) = 26.63, p_FDR_ = .000, partial η² = .32** |
|  | Max power | F(1, 56) = 2.59, p_FDR_ = .113, partial η² = .04 | F(1, 56) = 0.35, p_FDR_ = .557, partial η² = .01 |
|  | Max heel-strike force | F(1, 56) = 3.67, p_FDR_ = .076, partial η² = .06 | F(1, 56) = 4.19, p_FDR_ = .057, partial η² = .07 |
|  | Max toe-off force | F(1, 56) = 5.61, p_FDR_ = .053, partial η² = .09 | F(1, 56) = 4.94, p_FDR_ = .051, partial η² = .08 |
